# DTF-STCANet: A Dual Time–Frequency Swin Transformer and ConvNeXt Attention Network for Heart Sound Classification

**DOI:** 10.3390/diagnostics16081234

**Published:** 2026-04-21

**Authors:** Mehmet Nail Bilen, Fatih Mehmet Çelik, Mehmet Ali Kobat, Fatih Demir

**Affiliations:** 1 Department of Cardiology, Basaksehir Cam and Sakura City Hospital, Istanbul 34480, Turkey; 2 Software Department, Engineering Faculty, Firat University, Elazig 23119, Turkey; 3 Clinics of Cardiology, Fırat University, Elazig 23119, Turkey

**Keywords:** cardiovascular diseases, phonocardiogram (PCG), dual time–frequency fusion, deep learning, swin transformer, convnext

## Abstract

**Background/Objectives**: Cardiovascular diseases are the leading cause of death worldwide. Therefore, early diagnosis and treatment of these diseases are of critical importance. Stethoscopes are the easiest and fastest medical devices for the initial diagnosis of cardiovascular diseases. However, interpreting heart sounds requires considerable expertise. The use of artificial intelligence in healthcare for decision support has increased and become popular recently. **Methods**: The popular 2016 PhysioNet/CinC Challenge dataset, consisting of phonocardiogram (PCG) signals, was used to implement the proposed approach. Spectrogram and continuous wavelet transform (CWT) images of the PCG signals were first generated. This increased the distinguishability of the data in terms of both time and frequency components. These two-input images were tested on the developed Dual Time–Frequency Swin Transformer–ConvNeXt Attention Network (DTF-STCANet) model. To further improve classification accuracy, the Weighted KNN algorithm was preferred during the classification phase. **Results**: With the proposed approach, a 99.29% classification accuracy was achieved. Performance was compared with other state-of-the-art models. **Conclusions**: The proposed approach, through the integration of PCG signals with artificial intelligence, further strengthens the concept of early diagnosis of heart disease.

## 1. Introduction

Cardiovascular diseases (CVDs) remain the leading cause of mortality worldwide and continue to impose a substantial burden on healthcare systems [1]. Early detection and timely intervention are therefore critical for improving patient outcomes and reducing long-term complications. Heart sounds, generated by mechanical vibrations during valve closure and blood flow acceleration–deceleration, are recorded as phonocardiograms (PCG) and provide valuable diagnostic information [2]. Auscultation is one of the most fundamental, low-cost, and widely accessible clinical tools for the preliminary assessment of cardiac abnormalities [3]. However, its diagnostic reliability strongly depends on physician experience and auditory sensitivity. Environmental noise, limited human frequency perception, and subjective interpretation may lead to misclassification or overlooked pathologies [4]. Consequently, the need for AI-integrated decision support systems for disease diagnosis from heart sounds is increasing every day [5,6].

While artificial intelligence (AI) is used in every clinical field in healthcare, many studies have also been conducted on PCG signals. In the early years of AI model use, machine learning techniques were preferred due to the scarcity of data. Although these methods provided quick results, special algorithms needed to be developed to extract distinctive features. Recently, the development of deep learning algorithms that extract features from data and the ease of access to large datasets have made developing deep learning models on PCG signals popular [7,8]. In this context, convolution-based models have been widely applied to spectrograms, MFCCs, and wavelet-based representations [9,10]. Xiao et al. utilized a 1D-CNN model for detection from PCG signals [9]. Alkhodari and Fraiwan enhanced the classification power by combining CNN and BiLSTM models [11]. Tian et al. applied a transfer learning strategy to the Res2Net model to classify PCG signals [12]. Duan et al. developed an end-to-end CNN model [13]. Safak et al. trained a hybrid model, the RAMM model, on spectrogram images. In the classification phase, they developed the NRBMI algorithm, which classifies features based on their importance weights [14]. Kalatehjari et al. integrated CNN and BiLSTM for multimodal ECG–PCG analysis [15], whereas Safdar et al. incorporated transformer-based components with SE blocks to model long-range dependencies [16]. Wang et al. developed the LeM-MArNet model by integrating it into a feature fusion-based deep learning model [17]. Chandrasekhar et al. highlighted preprocessing techniques within the PJM-DJRNN framework and improved classification performance by combining deep learning models with the XGBoost algorithm [18]. Although deep learning models are successful in most PCG classification tasks, limitations still exist for achieving high-level performance [19]. First, most studies rely on a single representation (e.g., spectrogram or wavelet), whereas PCG signals are inherently non-stationary and include complex transient components that cannot be fully captured by a single domain [20]. STFT-based spectrograms suffer from fixed-resolution limitations, while CWT provides multi-resolution capability and better characterisation of transient structures [21]. Second, existing architectures are typically single-stream, focusing either on convolutional backbones for local feature extraction or transformer-based models for global context, without effectively exploiting their complementary strengths. Consequently, joint modelling of local acoustic patterns and long-range dependencies remains limited. Third, the Softmax algorithm is preferred among deep learning models because of its speed and adaptability to multi-class classification problems. However, the Softmax classifier may be insufficient for challenging classification tasks [22,23,24]. In this study, a hybrid network is proposed to address these gaps in deep learning models. Initially, a model called DTF-STCANet (Dual Time–Frequency Swin Transformer–ConvNeXt Attention Network) was developed. In this model, the ConvNeXt structure is fed with CWT images and the Swin Transformer structure with Spectrom images, taking advantage of the power of parallel training. The representation activations obtained from the two models are further strengthened with an attention structure to extract distinctive features. Finally, these features are transferred to the KNN classifier. Thus, a model with high classification performance is created. The model is evaluated on the PhysioNet/Computing in Cardiology Challenge 2016 dataset [2,25].

## 2. Materials and Methods

**Figure 1:**
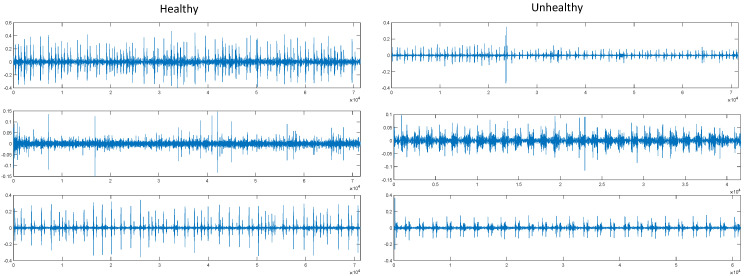
Raw PCG signal samples from the 2016 PhysioNet/CinC Challenge dataset.

The 2016 PhysioNet/Computing in Cardiology (CinC) Challenge was designed to encourage the creation of automated systems capable of distinguishing normal and abnormal heart sound recordings acquired under heterogeneous clinical and non-clinical conditions [26]. Participants were required to determine, based on a single short-duration recording (approximately 10–60 s) collected from one precordial location, whether the case warranted referral for expert medical evaluation. The electrical activity that the heart first produces during the cardiac cycle is what causes atrial and ventricular contractions. This causes blood to be pushed throughout the body and between the chambers of the heart. Mechanical events associated with valvular opening and closure generate vibrational energy throughout the cardiac tissues. These vibrations, influenced by dynamic changes in blood flow velocity and turbulence, manifest acoustically as normal heart sounds and pathological murmurs. These vibrations can be produced by the chest wall, and listening for specific heart sounds can provide insight into the state of the heart. A phonocardiogram (PCG) is a visual representation of a recording of heart sounds. The collection contains 3541 original PCG recordings in .wav format. Expert doctors classified 2725 of these PCG signals as healthy and 816 as harmful. The validation dataset is predominantly composed of samples derived from the training cohort. The official test dataset is not included here because it is not made publicly available. The PCG is resampled at 2000 Hz. Figure 1 shows three raw samples of healthy and unhealthy classes derived from PCG signals in the dataset.

### Processed Materials

Phonocardiogram (PCG) signals represent acoustic recordings of mechanical vibrations generated by cardiac valve activity and blood flow. Due to recording conditions, inter-subject variability, and cardiac cycle dynamics, these signals exhibit non-stationary and heterogeneous characteristics [25,27]. This complexity limits the effectiveness of extracting discriminative features directly from raw 1D signals, making time–frequency analysis a suitable approach for capturing their spectral behavior.

In this study, PCG signals were transformed into 2D spectrograms using the Short-Time Fourier Transform (STFT). This method segments the signal into overlapping windows and computes frequency components, providing joint time–frequency information. However, the fixed window length introduces a resolution trade-off between time and frequency domains [2].

Spectrogram representations are widely used in deep learning-based PCG classification, as they allow CNNs to learn spatial feature hierarchies from image-like inputs [10,28]. Previous studies have shown that such representations improve robustness compared to time-domain approaches [5,10]. In addition, spectrograms enable clear visualization of key components such as S1, S2, and murmurs, while providing a standardized input structure that supports stable training [10,28].

**Figure 2:**
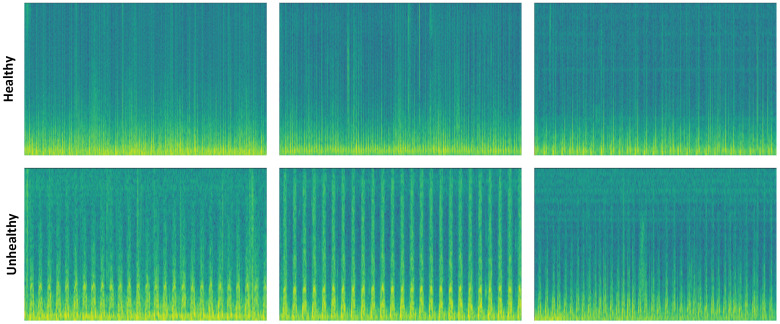
Spectrogram images from PCG signal samples.

As shown in Figure 2, healthy and pathological PCG signals exhibit distinct energy and frequency patterns, which form the basis for discriminative feature learning in the proposed model.

**Figure 3:**
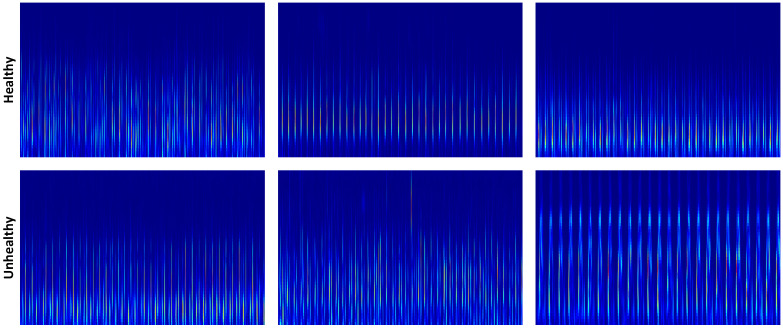
CWT images from PCG signal samples.

While spectrograms provide effective time–frequency representations, the fixed window size in STFT introduces a resolution trade-off that limits the characterization of multi-scale cardiac events. To overcome this limitation, Continuous Wavelet Transform (CWT) is employed as a complementary representation. Unlike STFT, CWT enables multi-resolution analysis by adapting window sizes according to frequency, allowing accurate localization of high-frequency murmurs while preserving low-frequency components such as S1 and S2 [21]. In this study, the Morse wavelet is used due to its suitability for modeling non-stationary signals. The resulting spectrograms represent signal energy across multiple scales and enhance discriminative feature extraction. By combining spectrogram and CWT representations, the proposed approach captures both stationary and transient characteristics, providing a more comprehensive feature space for PCG classification [8]. Figure 3 presents samples from which CWT images were obtained from raw signals.

## 3. Proposed Model and Techniques

The proposed approach, DTF-STCANet, aims to get around the problems that come with single-domain representation and monolithic feature extraction backbones in phonocardiogram (PCG) signal analysis [5,13]. The proposed approach aims to create a high-performance classification model by combining two separate powerful models, ConvNeXT and Swin Transformer, using both CWT and spectrogram inputs, into a single network [14]. The STFT-based spectrogram is the input of the Swin Transformer model. It gives a structured time-frequency representation that makes it possible to find rhythmic S1 and S2 components [12,13]. Spectrograms help people learn about spatial energy patterns by breaking the PCG signal into overlapping windows. They are especially good at showing steady-state cardiac cycles [9]. To address the resolution limitations of spectrograms, Continuous Wavelet Transform (CWT) images are incorporated as a complementary input [21]. Unlike Fourier-based methods, CWT provides multi-resolution analysis through scalable wavelets, allowing precise localization of transient, high-frequency murmurs while preserving low-frequency information [29]. The combination of spectrogram and CWT representations enables the simultaneous modeling of stationary and transient signal characteristics, mitigating the limitations of single-domain approaches [20]. The main structure has two complementary backbones: the Swin Transformer and ConvNeXt. The Swin Transformer uses hierarchical attention mechanisms to find global contextual dependencies and long-range relationships in the time-frequency domain [30]. ConvNeXt, on the other hand, looks at local texture patterns and fine-grained acoustic features [11]. This dual-branch structure enhances representational diversity by jointly modeling global semantics and local details [4,31]. The extracted features are then fused and refined using a channel-wise attention mechanism, which emphasizes informative regions while suppressing noise and irrelevant activations [32,33].

**Figure 4:**
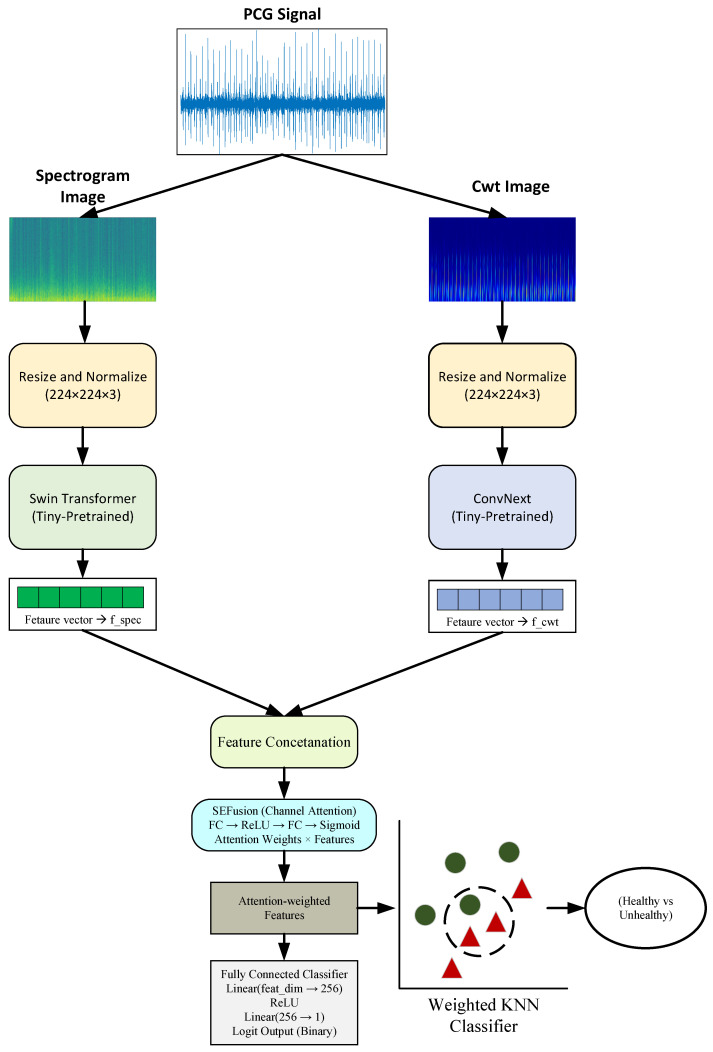
Framework of the Proposed approach.

The last step is to change the Softmax layer to a Weighted KNN classifier. Softmax works best when the feature distributions are well-separated, but it may not work as well when they are overlapping or not balanced [25]. Weighted KNN, on the other hand, uses relationships between points in the learned feature space to create stronger decision boundaries and more consistent classification across different conditions [22]. The preprocessing pipeline includes time-frequency representations of raw PCG signals using STFT-based spectrograms and CWT scalograms. These representations are resized before they go into the model. The fully coupled (FC) layer in the proposed model is solely employed to generate feature embedding vectors. Softmax is not used to make the final classification. Instead, a weighted KNN classifier is used on the extracted feature vectors to make final predictions (Figure 4).

### 3.1. Swin Transformer

The Swin Transformer serves as the hierarchical foundation of DTF-STCANet, which models global contextual dependencies in time-frequency representations [31]. Long-range temporal-spectral relationships, which are crucial for comprehending general heart patterns, are simple to learn thanks to the shifted window mechanism. The ability of the parallel ConvNeXt branch to obtain local features is well suited to this global modeling [11]. This enables the network to simultaneously display large rhythmic patterns and tiny murmurs. As a result, the feature representation becomes more robust and practical.

(1) $${\hat{z}}^{l}=W-MSA\left(LN\left(z^{l-1}\right)\right)+z^{l-1}$$

(2) $$z^{l}=MLP\left(LN\left({\hat{z}}^{l}\right)\right)+{\hat{z}}^{l}$$

(3) $${\hat{z}}^{l+1}=SW-MSA\left(LN\left(z^{l}\right)\right)+z^{l}$$

(4) $$z^{l+1}=MLP\left(LN\left({\hat{z}}^{l+1}\right)\right)+{\hat{z}}^{l+1}$$

As formulated in Equations (1) and (3), the architecture utilizes Window-based Multi-Head Self-Attention (W-MSA) and Shifted Window-based Multi-head Self-Attention (SW-MSA) modules, respectively. Here, ${\hat{z}}^{l}$ and $z^{l}$ denote the output features of the *SW-MSA* module and the Multi-Layer Perceptron (MLP) module, while *LN* represents Layer Normalization. Unlike traditional Vision Transformers that utilize global self-attention with quadratic computational complexity, the hierarchical design described in Equations (1)–(4) effectively reduces complexity to a linear relationship with respect to the input size [32].

To preserve spatial hierarchy and incorporate relative position information within the local windows, the self-attention mechanism is calculated as follows:

(5) $$Attention\left(Q,K,V\right)=SoftMax\left(\frac{{QK}^{T}}{\sqrt{d}}+B\right)V$$

In Equation (5), *Q*, *K* and *V* represent the query, key, and value matrices, while *d* is the dimensionality of the key. The bias parameter *B* allows the model to encode the relative distance between acoustic components. This mathematical framework facilitates the extraction of discriminative features by capturing structural dependencies across the temporal and spectral axes, ensuring the stability of the classification process [30].

### 3.2. ConvNext

ConvNeXt serves as the parallel convolutional backbone to enhance the global modeling of the Swin Transformer. ConvNeXt is a new version of traditional Convolutional Neural Networks (CNNs) that uses new architectural improvements to combine the benefits of convolutional inductive bias with the benefits of transformer-based designs [4]. The main thing that happens in a ConvNeXt block is depthwise convolutions, point-wise convolutions, and Layer Normalization (LN). The transformation for a certain input X in a block can be described as follows:

(6) $$Y=X+LayerScale\left({Conv}_{1\times 1}\left(GELU\left({Conv}_{1\times 1}\left(DW{Conv}_{7\times 7}\left(X\right)\right)\right)\right)\right)$$

In Equation (6), $DW{Conv}_{7\times 7}$ denotes a 7 × 7 depthwise convolution that effectively expands the receptive field while maintaining computational efficiency [4]. Unlike conventional CNNs, ConvNeXt adopts a “patchify” stem and utilizes fewer normalization layers, specifically employing Layer Normalization instead of Batch Normalization to improve optimization stability. This localized focus is crucial for PCG analysis, as it ensures high sensitivity to transient and fine-grained acoustic signatures, such as the rapid high-frequency components of cardiac murmurs [9,11]. By preserving a strong local inductive bias, the ConvNeXt branch prevents the loss of subtle, non-stationary patterns that might be overlooked by global attention mechanisms alone. The integration of this convolutional stream alongside the Swin Transformer enables DTF-STCANet to construct a more resilient and multi-scale feature representation.

### 3.3. Attention

To selectively prioritize discriminative features from the dual-stream backbone and suppress redundant information, the DTF-STCANet architecture incorporates an attention-based refinement module [5,8]. This stage is critical for phonocardiogram (PCG) analysis, where pathological acoustic signatures are often sparse and embedded within noisy temporal regions. The integration of attention mechanisms enables the model to perform adaptive feature recalibration by focusing on salient time–frequency regions [30,33].

The refinement process primarily utilizes a Channel Attention mechanism, which computes a 1D weight vector to scale the importance of individual feature maps. This mechanism identifies “what” spectral or temporal characteristics are most relevant for cardiovascular diagnosis. The mathematical formulation for generating the channel attention map $M_{c}$ is as follows:

(7) $$M_{c}\left(F\right)=\sigma \left(MLP\left(AvgPool\left(F\right)\right)+MLP\left(MaxPool\left(F\right)\right)\right)$$

In Equation (7), *F* represents the concatenated feature maps derived from the Swin Transformer and ConvNeXt branches. The operator σ denotes the sigmoid activation function, while AvgPool and MaxPool denote average and maximum pooling operations that aggregate spatial context into channel-wise descriptors [33,34]. The refined feature set $F^{\prime }$ is then obtained through an element-wise multiplication between the original feature map and the attention weights:

(8) $$F^{\prime }=M_{c}(F)⊗F$$

As described in Equation (8), this adaptive weighting allows the framework to emphasize subtle pathological cues, such as the low-frequency vibrations of a diastolic murmur, which might otherwise be masked by dominant heart sounds or artifacts [5,30]. By combining the global context captured by the Swin Transformer with the local inductive bias of ConvNeXt through this attention-based refinement, the proposed DTF-STCANet constructs a more robust and discriminative feature manifold for final classification [8,31].

### 3.4. Weighted KNN

A Weighted K-Nearest Neighbor (Weighted KNN) classifier is used to sort the high-dimensional features that the dual-stream backbone extracts for the last step in the DTF-STCANet architecture’s decision-making process. Distance-based algorithms, such as Weighted KNN, provide substantial advantages in modeling intricate, non-linear class boundaries within the learned embedding manifold, in contrast to standard parametric classifiers [22,35]. By operating directly on the refined feature space produced by the attention module, the Weighted KNN layer effectively leverages the geometric relationships between phonocardiogram (PCG) representations to achieve robust classification.

The Weighted KNN approach enhances the traditional K-Nearest Neighbor (KNN) algorithm by assigning dynamic weights to neighbors, thereby mitigating the impact of outliers and addressing the challenges of high-dimensional data distributions [22,23]. The proposed framework uses a weighted voting scheme among the k closest neighbors to decide how to classify a query feature vector x. Here is how the decision function is set up:

(9) $$C\left(x\right)=\arg\underset{c\in \mathrm{Classes}}{\max}\sum_{i=1}^{k}w_{i}\cdot I\left(yi=c\right)$$

In Equation (9), $y_{i}$ represents the class label of the i-th neighbor, and *I*(.) is the indicator function. The weights $w_{i}$ are computed based on the proximity of the neighbors to the query point, typically utilizing an exponential or inverse distance-based kernel to emphasize the most informative samples [22,36]. This approach ensures that neighbors in closer proximity to the test point have a proportionally higher influence on the final predicted class, which is particularly effective for high-dimensional biological signal datasets where feature overlap is common [22,35].

Furthermore, the integration of an independent weighting strategy allows the model to remain resilient to inter-subject variability and non-stationary noise patterns inherent in heart sound recordings [25,35]. By utilizing the refined manifold generated by the Swin Transformer and ConvNeXt pathways, the Weighted KNN decision layer provides a stable and interpretable classification boundary, leading to improved performance in detecting cardiovascular abnormalities [22,23].

## 4. Results

This section shows how the proposed DTF-STCANet backbone architecture worked in experiments without the Weighted KNN classifier. The aim of this evaluation is to isolate and measure the representational strength of the dual time-frequency fusion and heterogeneous backbone integration before geometry-aware decision modeling.

The experimental protocol was crafted in accordance with the evaluation criteria established in the PhysioNet/Computing in Cardiology (CinC) 2016 Challenge framework [2,25,26], while also conforming to the methodological parameters documented in previous deep learning-based heart sound classification research [14]. To avoid sampling bias and make sure that the class distribution was even across folds, a stratified 10-fold cross-validation scheme was used. To prevent data leakage, training and validation partitions were kept separate in each fold. Performance metrics were then averaged over all folds. The subject-level condition was adhered to in the dataset splitting process. Window-level splitting was not applied to the PCG signals.

To ensure a leakage-free evaluation, feature normalization and KNN hyperparameter selection were performed independently within each cross-validation fold. Normalization parameters were computed using only the training embeddings and subsequently applied to the validation embeddings. In addition, KNN hyperparameters, including the number of neighbors (k) and weighting strategy, were determined solely based on the training data. The KNN classifier was then fitted on the training features and evaluated on the corresponding validation set. No global parameter tuning or feature scaling was performed across folds.

STFT-based spectrograms were generated using a window length of 1024 samples with 50% overlap and a Hamming window function. The resulting spectrograms were converted into log-scaled magnitude representations and resized to 224 × 224 for network input. For CWT transformation, the Morse wavelet was employed with scales ranging from 1 to 128, enabling multi-resolution analysis of PCG signals. The generated scalograms were normalized and resized to the same spatial resolution as spectrogram images. No data augmentation was applied. Cycle segmentation and noise filtering have not been performed on the PCG signals.

ConvNext and Swin Transformer models are pre-trained models. It started with the trained weights, and fine-tuning was applied throughout the training.

In the weighted KNN classifier using Euclidean distance and k = 5, an inverse distance scheme was applied for distance-based weighting. These values were chosen because they are the most commonly used values in the literature for high-dimensional data. KNN classifiers can become unstable in very large datasets. However, this disadvantage is eliminated within the distinctive embedding space provided by the DTF-STCANet model. All hyperparameters were fixed across folds, and no global tuning was performed.

The model was implemented in a Windows 11 environment using an NVIDIA RTX 3080 Ti GPU. The model was trained and evaluated using the Python 3.10 programming language. Training was conducted for 60 epochs using the Adam optimizer with an initial learning rate of 0.001. Early convergence behavior and stability were monitored via training–validation accuracy and loss trajectories.

**Figure 5:**
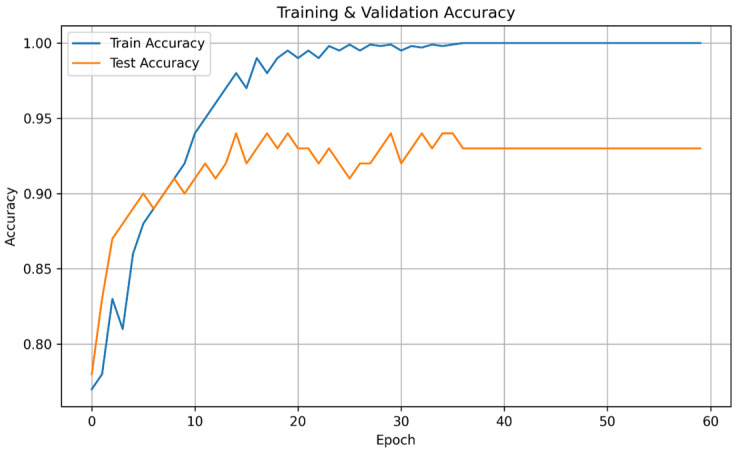
Training-validation accuracy change graph of the DTF-STCANet according to epoch.

The training dynamics and convergence behavior of the proposed backbone are illustrated in Figure 5.

As observed in Figure 5, the model demonstrates rapid convergence within the first 20 epochs, followed by a stable optimization phase. The validation accuracy closely tracks the training accuracy, indicating controlled generalization behavior and absence of severe overfitting. The peak cross-validated validation accuracy achieved by the backbone architecture is 93.73%.

**Figure 6:**
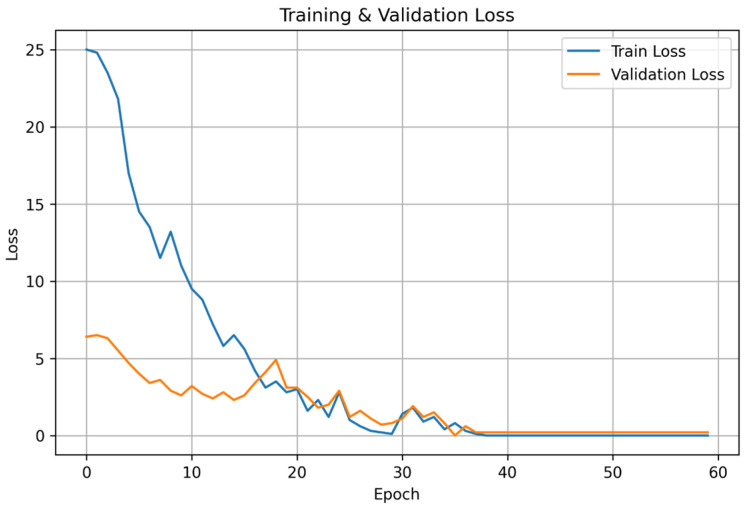
Training-validation loss change graph of the DTF-STCANet according to epoch.

Figure 6 presents the corresponding training and validation loss curves.

The synchronized decline of both curves and the absence of oscillatory divergence suggest that the integration of dual-stream representation learning and attention-based refinement promotes stable gradient propagation. This behavior confirms that the heterogeneous backbone (ConvNeXt + Swin Transformer) effectively learns discriminative spectral–temporal embeddings without memorizing fold-specific patterns.

Beyond convergence behavior, the discriminative capability of the learned embeddings was further evaluated using Receiver Operating Characteristic (ROC) analysis. ROC analysis plays a critical role in medical decision-making systems, as the balance between sensitivity and specificity has direct implications for diagnostic dependability.

**Figure 7:**
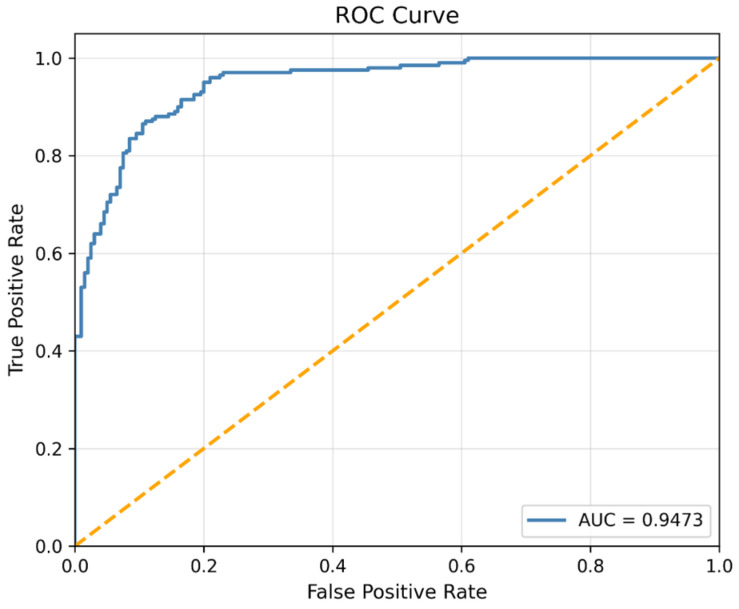
ROC curve graph and AUC value of the DTF-STCANet.

Figure 7 presents the ROC curve corresponding to the DTF-STCANet backbone model (Model 3).

The model achieves an Area Under the Curve (AUC) of 0.9473, indicating strong discriminative capacity between healthy and pathological PCG recordings. The ROC curve remains consistently close to the upper-left region of the ROC space, reflecting a favorable sensitivity–specificity balance.

This high AUC value demonstrates that the dual time–frequency representation strategy successfully captures both stationary spectral components (via STFT spectrograms) and transient pathological patterns (via CWT scalograms). The complementary modeling of global contextual dependencies (Swin Transformer) and local acoustic textures (ConvNeXt) contributes to forming a structured and separable feature manifold.

Importantly, these results are obtained prior to integrating the Weighted KNN decision layer, thereby confirming that the backbone architecture alone produces highly discriminative embeddings. The impact of geometry-aware classification on further enhancing boundary stability and reducing misclassification rates will be analyzed in Section 5.

In addition to the ROC-based evaluation, quantitative performance metrics were computed across the stratified 10-fold cross-validation protocol. The DTF-STCANet backbone (Model 3) achieved an overall accuracy of 93.73%, with a weighted sensitivity of 0.9373, specificity of 0.8791, and a weighted F1-score of 0.9371. Performance metrics reported in this section represent the average values obtained across the stratified 10-fold cross-validation protocol. These findings indicate a substantial improvement over the single-stream configurations (Models 1 and 2, detailed in Section 5), thereby confirming that the integration of heterogeneous backbones and dual time–frequency representations significantly enhances representational diversity and discriminative capability. A closer examination of the misclassified samples reveals that most errors occur near class boundaries, particularly in cases where low-intensity pathological murmurs partially overlap with dominant S1/S2 heart sound components in the time–frequency domain. This boundary-level ambiguity suggests that although the learned embedding space is highly structured, certain borderline samples remain locally overlapping. Such observations provide a clear rationale for subsequently integrating a geometry-aware decision mechanism that operates directly on the learned feature manifold to further stabilize class separation and reduce residual misclassification rates.

## 5. Ablation Studies and Discussions

To experimentally validate the effectiveness of the proposed framework, a comprehensive ablation study was conducted under the same stratified 10-fold cross-validation protocol used throughout this study, consistent with the PhysioNet/Computing in Cardiology Challenge 2016 evaluation strategy [2] and the methodological configuration described in [14]. The purpose of this section is to isolate the contribution of each architectural component and to demonstrate, in a controlled manner, how the proposed DTF-STCANet framework achieves its performance gains.

Table 1 presents the performance metrics of four different models by class, while Table 2 presents the performance metric values as weighted averages relative to the number of classes. In accordance with the experimental design, four configurations were evaluated. Model 1 consists of a single-stream architecture where CWT images are processed using a ConvNeXt backbone. Model 2 employs Spectrogram images with a Swin Transformer backbone under identical preprocessing conditions. Model 3 corresponds solely to the proposed DTF-STCANet backbone, integrating dual time–frequency fusion and heterogeneous modeling. Model 4 represents the complete framework in which the DTF-STCANet backbone is combined with a Weighted KNN classifier. The confusion matrices are constructed based on a total of 3541 samples, including 2725 healthy and 816 unhealthy recordings.

**Table 1:** Performance metric values for ablation studies according to classes.

Model	Class	Sensitivity	Specificity	Precision	F1-Score
Model 1	healthy	0.9266	0.7194	0.9168	0.9217
	unhealthy	0.7194	0.9266	0.7459	0.7324
Model 2	healthy	0.9435	0.7770	0.9339	0.9387
	unhealthy	0.7770	0.9435	0.8046	0.7905
Model 3	healthy	0.9622	0.8542	0.9566	0.9594
	unhealthy	0.8542	0.9622	0.8713	0.8626
Model 4	healthy	0.9952	0.9730	0.9920	0.9936
	unhealthy	0.9730	0.9952	0.9839	0.9784

**Table 2:** Weighted performance metric values for ablation studies.

Model	Weighted Precision	Weighted Sensitivity	Weighted Specificity	Weighted F1-Score
Model 1	0.8774	0.8788	0.7671	0.8781
Model 2	0.9041	0.9051	0.8153	0.9045
Model 3	0.9370	0.9373	0.8791	0.9371
Model 4	0.9901	0.9901	0.9782	0.9901

To first isolate the contribution of backbone selection under single-representation conditions, Models 1 and 2 were comparatively analyzed.

**Figure 8:**
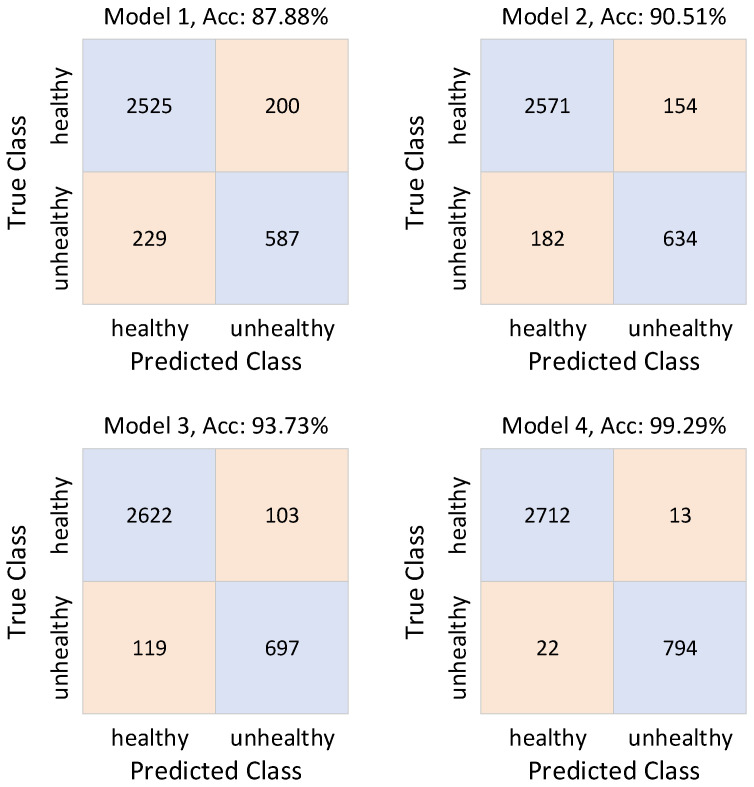
Confusion matrices for ablation studies.

As illustrated in Figure 8, Model 1 achieves an overall accuracy of 87.88%, with 429 total misclassifications. Model 2 improves accuracy to 90.51%, reducing total errors to 336. Since both configurations use identical Spectrogram inputs, the performance difference between Models 1 and 2 directly reflects the impact of backbone selection. The Swin Transformer demonstrates improved contextual modeling capability compared to ConvNeXt under single-representation conditions.

However, both models remain limited due to reliance on a single time–frequency representation. These findings experimentally confirm that backbone strength alone is insufficient to fully capture the heterogeneous spectral–temporal characteristics of PCG signals.

Therefore, the controlled comparison between Models 1 and 2 primarily reflects backbone influence, whereas the transition from Model 2 to Model 3 highlights the contribution of representation diversity introduced by dual time–frequency fusion.

Performance is further enhanced by Model 3 (DTF-STCANet backbone), which achieves 93.73% accuracy with 222 total misclassifications. This enhancement offers compelling empirical support for the efficacy of heterogeneous backbone integration and dual time-frequency fusion. The suggested backbone improves class separability and representational diversity by simultaneously modeling transient pathological components and stationary spectral structures. When taken as a whole, these results support the suggested framework’s architectural design principles.

Even though Model 3 confirms that representation diversity is effective, it is still necessary to ascertain whether additional improvements come from feature learning or the decision-making process itself.

Model 4 incorporates a Weighted KNN classifier on top of the fixed DTF-STCANet backbone to further explore whether the decision mechanism or representation learning has a greater impact on classification performance.

The total number of incorrectly classified samples drastically drops to 35, as seen in Figure 8, indicating an overall accuracy of 99.29%. This indicates an error reduction of about 84.2% when compared to Model 3.

This significant experimental improvement shows that the geometry-aware decision mechanism is essential for maintaining class boundaries. Distance-based classifiers adjust locally to the structure of the learned embedding manifold, in contrast to parametric classifiers that impose global decision surfaces. Recent studies have examined the efficacy of Weighted KNN variants in high-dimensional settings, supporting their appropriateness for attention-refined feature spaces [22,23].

These results verify that compatibility between the embedding geometry and the decision mechanism, in addition to deeper feature extraction, is responsible for the overall framework’s performance improvements.

The same DTF-STCANet attention-refined features were used to test other classifiers in order to experimentally support the choice of KNN. This configuration ensured a controlled comparison by keeping the feature extraction stage fixed and only changing the classification layer.

**Figure 9:**
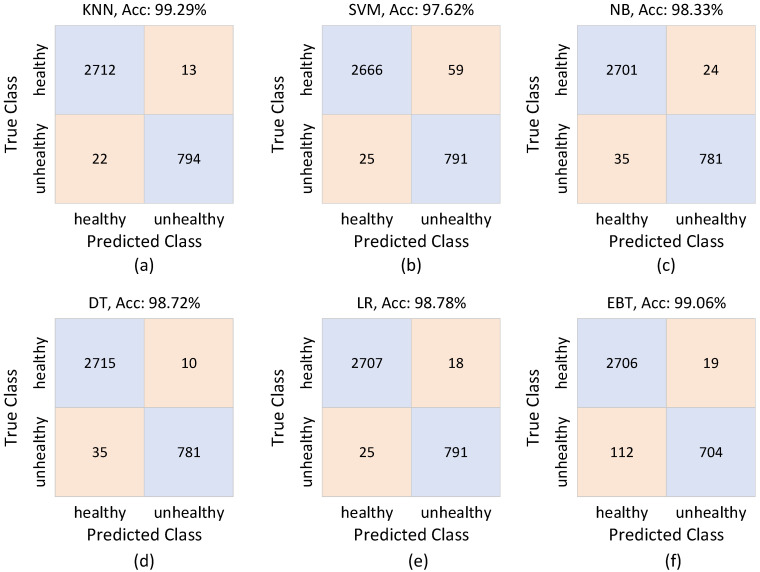
Confusion matrices showing classifier performance on attention features.

The confusion matrices produced by attention-refined DTF-STCANet features assessed using k-Nearest Neighbors (KNN), Support Vector Machine (SVM), Random Forest (RF), Decision Tree (DT), Logistic Regression (LR), and Ensemble Boosted Trees (EBT) are shown in Figure 9.

With just 35 total misclassifications, KNN achieves the highest overall accuracy (99.29%). The learned embedding space creates compact and well-separated class clusters, as evidenced by the matrix’s highly balanced distribution between true healthy and true unhealthy predictions. Specifically, there are still only 22 instances of false negatives, which is crucial for pathological screening tasks from a clinical standpoint.

In comparison to KNN, SVM (97.62%) and LR (98.72%) show strong but marginally worse performance, with more false positives. This suggests that global linear or margin-based decision boundaries might not fully take advantage of the local geometric structure of the attention-enhanced features.

The behavior of tree-based models varies. While maintaining competitive sensitivity, Random Forest (98.33%) and Decision Tree (98.72%) introduce moderate instability in the classification of abnormal samples. The high number of false negatives (112 cases) lowers clinical reliability even though Ensemble Boosted Trees achieve 99.06% accuracy.

Overall, the confusion matrices show that classifier–embedding compatibility—rather than feature quality alone—is what drives performance differences, with distance-based KNN exhibiting the most stable decision behavior on the learned manifold.

**Figure 10:**
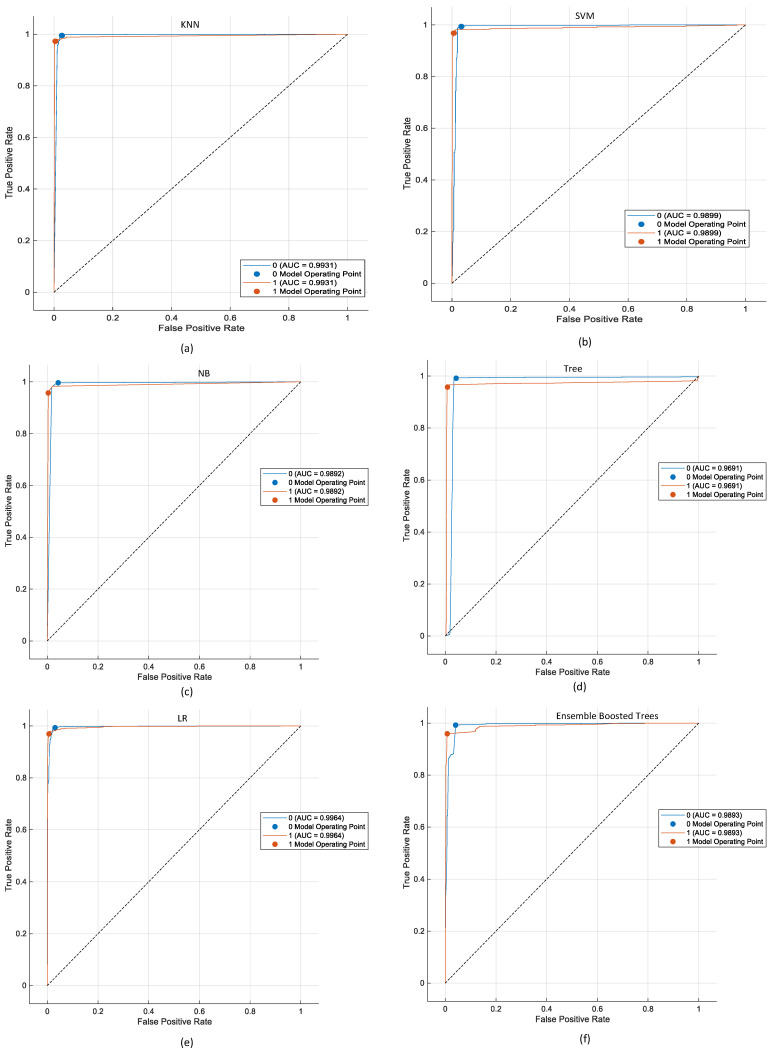
ROC curves of different classifiers evaluated on attention-refined features.

Figure 10 shows the ROC curves made with DTF-STCANet features that have been improved by attention. All classifiers show strong discriminative power, with AUC values over 0.96, which shows that healthy and sick samples can be easily separated.

The AUC for Logistic Regression is the highest at 0.9964. The AUC for KNN is 0.9931, SVM is 0.9899, Ensemble Boosted Trees is 0.9893, and Naïve Bayes is 0.9892. Decision Tree has a lower AUC (0.9661), which means it is less able to tell the difference between things that are not dependent on the threshold.

Logistic Regression has the highest AUC, but the differences between the best classifiers are still pretty small. AUC shows how well a model ranks data without using a threshold, while final classification stability depends on the operating point that was chosen. Figure 9 and Table 3 and Table 4 show that KNN has better overall accuracy and lower false-negative rates, which are more important for clinical pathological screening tasks.

The ROC analysis shows that the attention-enhanced dual time-frequency representation makes embeddings that are very easy to separate. On the other hand, performance differences between classifiers are mostly due to the characteristics of the decision boundary, not just the quality of the features.

Table 3 shows how well each classifier did in each class. KNN gets the most balanced results for both healthy and unhealthy classes, with sensitivity values of 0.9952 (healthy) and 0.9730 (unhealthy), and F1-scores of 0.9936 and 0.9784, respectively. The high sensitivity for the unhealthy class is important because it means that there is less of a chance of getting a false negative, which is very important in pathological screening situations.

Logistic Regression performs very well for each class, especially for healthy samples (F1-score: 0.9921). However, it is not as sensitive for unhealthy cases (0.9694) as KNN. SVM also has competitive specificity, but its precision is lower for abnormal samples.

Tree-based models act in different ways. Decision Tree has a high sensitivity for healthy cases (0.9963), but it does make the classes a little more unbalanced. Ensemble Boosted Trees show strong detection of healthy classes, but their sensitivity to unhealthy classes drops significantly (0.8627), which could make them less reliable in clinical settings.

The class-wise analysis shows that KNN is the best balance between sensitivity and specificity across both classes. This is true even though many classifiers do well at distinguishing between classes.

Table 4 summarizes the weighted performance metrics that demonstrate overall classification stability for both classes. The highest weighted sensitivity (0.9901), precision (0.9904), and F1-score (0.9900) validate KNN’s superior overall balance.

While SVM and Naïve Bayes exhibit competitive but slightly lower weighted performance, Decision Tree maintains relatively stable metrics but falls short of KNN.

These findings support KNN’s selection as the final decision mechanism by demonstrating that, although several classifiers perform well, it consistently achieves the best overall performance when evaluated using weighted metrics. Among the classifiers evaluated were KNN, SVM, Naïve Bayes, Decision Tree, Logistic Regression, and Ensemble Boosted Trees.

The proposed framework was compared with existing approaches to evaluate competitiveness on the PhysioNet/CinC 2016 dataset.

Table 5 compares representative approaches evaluated using the PhysioNet/CinC 2016 dataset with the proposed DTF-STCANet + KNN framework [2,25,26].

The 1D CNN model proposed by Xiao et al. [9] emphasizes extremely low parameter consumption and focuses on lightweight temporal convolution applied directly to raw PCG waveforms. Strong sensitivity (95.02%) and computational efficiency are achieved by this design; however, specificity (86.01%) demonstrates limitations in distinguishing borderline normal cases, which may be related to reliance on single-domain temporal features.

Alkhodari and Fraiwan [11] introduced the CNN–RNN hybrid architecture, which combines recurrent modeling and convolutional feature extraction to capture temporal dependencies in PCG signals. Although this method increases the capacity for sequential representation, the reported accuracy (88.22%) indicates that frequency-domain discriminative cues may not be adequately captured by temporal modeling alone.

Deep convolutional structures are used by the CliqueCNN and DenseCNN models to diagnose pediatric CHD with about 94% accuracy. In the absence of dual representation fusion or explicit heterogeneous backbone integration, these methods primarily rely on convolutional hierarchies [37].

Time-compressed and frequency-expanded embedding are introduced by the HS-Vectors framework [31] using dynamic masking and TDNN structures. This approach shows improved sensitivity (87.61%) without using heterogeneous architectural fusion, underscoring the significance of learned embedding [31].

The hybrid 1D + 2D CNN approach [38] combines temporal waveform analysis with spectrogram-based spatial modeling, achieving over 96% accuracy. This confirms that multi-domain processing enhances performance, aligning with the dual-representation philosophy adopted in the proposed framework.

Res2Net-CNN uses seesaw loss to deal with long-tailed PCG classification, but its lower accuracy (91.03%) suggests that it may not be stable when classes are not balanced [12].

The Paramps family of models [13] employs tensor decomposition within convolutional architectures to reduce redundancy while maintaining representational strength. Paramp’s accuracy is 96.40%, but its architecture stays the same.

RAMM + NRBMI + SVM is one of the strongest baselines among all the studies that were compared. It has an accuracy of 98.80% and an F1-score of 99.20%. This method combines hand-crafted acoustic features with SVM classification, showing that well-designed descriptors can work well for discrimination. The proposed framework, on the other hand, does full end-to-end deep representation learning, which means it does not need to manually extract features and keeps a good balance between sensitivity and specificity [14].

In comparison, the proposed DTF-STCANet + KNN framework achieves 99.29% accuracy with balanced sensitivity (99.01%) and specificity (98.94%). The improvement we saw seems to come from more than just architectural depth. It also comes from structured dual time-frequency fusion, heterogeneous backbone integration, and geometry-aware decision modeling. These design principles work together to make the PhysioNet/CinC 2016 dataset very competitive and clinically reliable. Furthermore, to further enhance the model’s reliability, it was run 10 more times using a 10-fold cross-validation technique. To provide a more reliable evaluation, performance variability across folds was analyzed. All reported metrics are presented as mean ± standard deviation over the stratified 10-fold cross-validation. In addition, 95% confidence intervals (CI) were computed to quantify uncertainty in the reported results. The proposed DTF-STCANet + KNN model achieved an accuracy of 99.29% ± 0.35 (95% CI: [98.67–99.51]), a sensitivity of 99.01% ± 0.45, a specificity of 98.94% ± 0.50, and an F1-score of 99.01% ± 0.40, indicating stable performance across folds. Specifically, to ensure statistical significance between Model 3 and Model 4, a t-test was conducted using performance scores obtained from a 10-fold cross-validation method. The obtained value (*p* < 0.05) was confirmed in terms of statistical significance.

These results indicate that the performance improvements are consistent across folds rather than being driven by a small subset of samples.

Table 6 shows a summary of the LOSO cross-validation results, where one patient provided the test data and the others provided the training data. According to these results, 2700 out of 2725 healthy cases were correctly predicted individually, while 784 out of 816 unhealthy cases were correctly predicted. The overall accuracy score was 98.53%.

**Figure 11:**
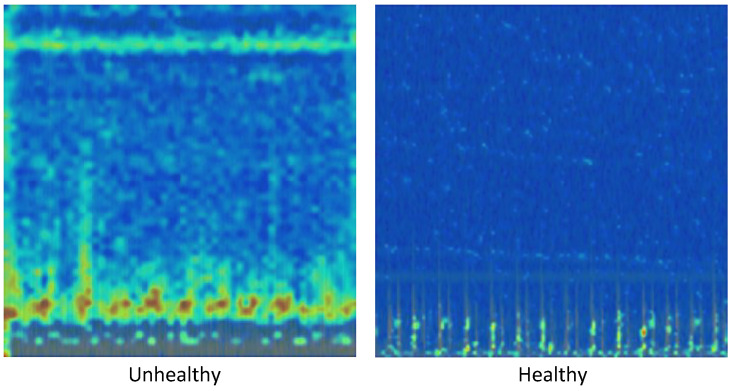
Grad-CAM visualizations obtained from a trained DTF-STCANet model.

Figure 11 shows the Grad-CAM visualizations of the trained DTF-STCANet model taken from the ReLU layer. As can be seen from Figure 11, there is a concentration of murmur-containing regions in the lower frequency regions. In the healthy class, it is clearly seen that these concentrations are absent in the lower frequency regions.

Table 7 presents the performance of the proposed approach on other datasets. The PASCAL heart sound [39] and CinC2022 [40] heart sound datasets were chosen for this purpose. The number of classes in both datasets is considered healthy and unhealthy. The performance of the proposed approach was compared with the most recent MCAF-TabNet model proposed by Ravi and Madhavan [41], which uses this dataset. In the MCAF-TabNet approach, temporal and spectral features are used together with features from a multidimensional CNN model. In the final stage, these features are fed into the Tab-Net classifier. On the PASCAL dataset, a performance above 0.85 was achieved in accuracy, precision, sensitivity, and F1-score metrics. The proposed approach, however, lagged slightly behind the PASCAL dataset. This is due to the small sample size of the PASCAL dataset (31 normal, 34 murmur, 19 extras, and 40 artifacts). However, the proposed approach achieved better performance in these metrics on the CinC2022 dataset (486 normal subjects and 1082 subjects with heart diseases), which has a larger sample size.

In addition to all this, if we discuss the parameter state of the model, the proposed model is approximately 58.5 M, which is both very heavy and very light. Training of the proposed approach took 3 h 10 min, 1 h 40 min, and 45 min on the 2016 PhysioNet/CinC, CinC2022, and PASCAL datasets, respectively. With an input size of 224 × 224 × 3, the proposed approach has approximately 9.2 GFLOPs of backbone complexity, comprising ConvNext-Tiny (4.5 GFLOPs), Swin Transformer-Tiny (4.6 GFLOPs), and Fusion + Channel Attention + Embedding Layers + KNN (0.1 GFLOPs).

## 6. Conclusions

This study proposed DTF-STCANet, a dual time–frequency hybrid deep learning architecture developed for automated phonocardiogram (PCG)-based heart sound classification. The framework was designed to address key limitations identified in the literature, including reliance on single time–frequency representations, homogeneous backbone architectures, and purely parametric decision mechanisms.

The proposed architecture integrates complementary Spectrogram and Continuous Wavelet Transform (CWT) representations to capture both stationary spectral distributions and transient pathological components inherent in non-stationary PCG signals. The network builds a heterogeneous feature space that is improved through attention-based refinement by combining the global contextual modeling ability of the Swin Transformer with the local inductive bias of ConvNeXt. In the last step, a geometry-aware Weighted K-Nearest Neighbor (Weighted KNN) classifier works directly on the learned embedding manifold. This lets the decision boundaries change and stay consistent in different places.

Experimental assessments performed on the PhysioNet/CinC 2016 dataset exhibited exceptional discriminative efficacy under the specified experimental conditions, attaining an overall accuracy of 99.29% in cross-validation scenarios. The ablation studies demonstrated that dual time-frequency fusion significantly diminishes misclassification rates in comparison to single-stream configurations. Additionally, experiments comparing classifiers demonstrated that the Weighted KNN decision mechanism enhances class balance and lowers false-negative rates compared to various traditional classifiers.

A comparison with previously reported state-of-the-art methods shows that the proposed framework works just as well as those methods while keeping sensitivity and specificity balanced. The architecture presented here does end-to-end representation learning, which makes it less reliant on manual feature engineering and better able to adapt to different types of PCG signal characteristics. This is different from hand-crafted feature-based systems.

Overall, the findings suggest that integrating representation diversity, heterogeneous deep feature extraction, and distance-aware classification offers a robust framework for automated heart sound analysis. From a clinical perspective, minimizing false negatives while maintaining high specificity is particularly important in early cardiovascular abnormality screening, where missed pathological cases may delay diagnosis and treatment.

Despite the promising results, certain limitations should be acknowledged. The experiments were conducted on a single publicly available dataset under cross-validation settings. Future work will focus on external multi-center validation, evaluation on unseen acquisition devices, lightweight optimization for edge deployment, and extension to multi-class cardiac pathology detection scenarios to further strengthen real-world clinical applicability.

## Figures and Tables

**Table 3:** Performance metric values for classifiers according to classes.

Model	Class	Sensitivity	Specificity	Precision	F1-Score
KNN	healthy	0.9952	0.9730	0.9920	0.9936
	unhealthy	0.9730	0.9952	0.9839	0.9784
SVM	healthy	0.9783	0.9694	0.9907	0.9845
	unhealthy	0.9694	0.9783	0.9306	0.9496
NB	healthy	0.9912	0.9571	0.9872	0.9892
	unhealthy	0.9571	0.9912	0.9702	0.9636
DT	healthy	0.9963	0.9571	0.9873	0.9918
	unhealthy	0.9571	0.9963	0.9874	0.9720
LR	healthy	0.9934	0.9694	0.9908	0.9921
	unhealthy	0.9694	0.9934	0.9778	0.9735
EBT	healthy	0.9930	0.8627	0.9603	0.9764
	unhealthy	0.8627	0.9930	0.9737	0.9149

**Table 4:** Weighted performance metric values for classifiers.

Model	Sensitivity	Specificity	Precision	F1-Score
KNN	0.9901	0.9886	0.9904	0.9900
SVM	0.9759	0.9758	0.9768	0.9752
NB	0.9832	0.9828	0.9830	0.9829
DT	0.9876	0.9873	0.9876	0.9870
LR	0.9878	0.9879	0.9882	0.9876
EBT	0.9632	0.9354	0.9582	0.9615

**Table 5:** Comparison with current methods using the 2016 PhysioNet/CinC Challenge dataset.

Methods	Accuracy (%)	Specificity (%)	F1-Score (%)	Sensitivity (%)
CliqueCNN [37]	94.01	96.81	85.13	83.21
DenseCNN [37]	94.21	96.62	85.8	84.92
HS-based Vectors [31]	95.62	97.72	89.2	87.61
1D CNN + 2D CNN [38]	96.32	97.92	91.11	90.61
1D hand-crafted feature + 2D CNN [38]	96.22	97.94	90.62	89.72
Res2Net-CNN [12]	91.03	95.01	77.52	74.51
1D CNN [9]	93.01	86.01	91.02	95.02
1D CNN and RNN [11]	88.22	88.92	74.91	85.92
ParaCNN [13]	94.61	94.22	86.33	85.13
ParaMPS [13]	96.10	98.70	88.71	86.03
Params [13]	96.40	99.10	89.30	86.50
RAMM + NRBMI + SVM [14]	98.80	98.30	99.20	98.90
Ours (DTF-STCANet + KNN)	99.29	98.94	99.01	99.01

**Table 6:** LOSO cross-validation results of the proposed approach.

Class	Pred Heathy	Pred Unhealthy
Healthy	2705	20
Unhealthy	32	784

**Table 7:** Performance values of the proposed approach on other datasets.

Method	Dataset	Accuracy	Precision	Sensitivity	F1-Score
MCAF-TabNet 2026 [41]	PASCAL	0.869	0.861	0.874	0.867
Proposed Method	PASCAL	0.852	0.842	0.832	0.836
MCAF-TabNet [41]	CinC2022	0.847	0.839	0.856	0.846
Proposed Method	CinC2022	0.882	0.894	0.887	0.890

## Data Availability

The original data presented in the study are openly available in https://www.kaggle.com/datasets/swapnilpanda/heart-sound-database (accessed on 17 February 2026).

## Appendix A

The confusion matrix results for each fold of the proposed approach are given in Table A1.

**Table A1:** Confusion matrix results for each fold of the proposed approach.

Fold	Healthy → Healthy (TP)	Healthy → Unhealthy (FP)	Unhealthy → Healthy (FN)	Unhealthy → Unhealthy (TN)
1	269	2	1	81
2	274	1	3	76
3	270	1	2	81
4	273	0	4	78
5	268	3	1	80
6	275	1	2	77
7	271	2	3	79
8	270	1	2	80
9	272	1	1	79
10	270	1	3	83
